# Aerial surveys for Antarctic minke whales (*Balaenoptera bonaerensis*) reveal sea ice dependent distribution patterns

**DOI:** 10.1002/ece3.5149

**Published:** 2019-04-30

**Authors:** Helena Herr, Natalie Kelly, Boris Dorschel, Marcus Huntemann, Karl‐Hermann Kock, Linn Sophia Lehnert, Ursula Siebert, Sacha Viquerat, Rob Williams, Meike Scheidat

**Affiliations:** ^1^ Institute for Terrestrial and Aquatic Wildlife Research University of Veterinary Medicine Hannover, Foundation Buesum Germany; ^2^ CSIRO Mathematical and Information Sciences Hobart Tasmania Australia; ^3^ Alfred Wegener Institute Helmholtz Centre for Polar and Marine Research Bremerhaven Germany; ^4^ Institute of Environmental Physics University of Bremen Bremen Germany; ^5^ von Thünen Institute Institute of Sea Fisheries Bremerhaven Germany; ^6^ Pew Fellow in Marine Conservation, Oceans Initiative Seattle Washington; ^7^ Wageningen Marine Research Wageningen University and Research IJmuiden The Netherlands; ^8^ Present address: Center of Natural History (CeNak) University of Hamburg Hamburg Germany; ^9^ Present address: Australian Antarctic Division Kingston Tasmania Australia; ^10^ Present address: Kiefernweg 11a 22949 Ammersbek Germany; ^11^ Present address: Leibniz Institute for Zoo and Wildlife Research (IZW) Berlin Germany; ^12^ Present address: Center of Natural History (CeNak) University of Hamburg Hamburg Germany

**Keywords:** Antarctic minke whale distribution, density surface models, distance sampling, marginal ice zone, ship‐based helicopter surveys, Southern Ocean

## Abstract

This study investigates the distribution of Antarctic minke whales (AMW) in relation to sea ice concentration and variations therein. Information on AMW densities in the sea ice‐covered parts of the Southern Ocean is required to contextualize abundance estimates obtained from circumpolar shipboard surveys in open waters, suggesting a 30% decline in AMW abundance. Conventional line‐transect shipboard surveys for density estimation are impossible in ice‐covered regions, therefore we used icebreaker‐supported helicopter surveys to obtain information on AMW densities along gradients of 0%–100% of ice concentration. We conducted five helicopter surveys in the Southern Ocean, between 2006 and 2013. Distance sampling data, satellite‐derived sea‐ice data, and bathymetric parameters were used in generalized additive models (GAMs) to produce predictions on how the density of AMWs varied over space and time, and with environmental covariates. Ice concentration, distance to the ice edge and distance from the shelf break were found to describe the distribution of AMWs. Highest densities were predicted at the ice edge and through to medium ice concentrations. Medium densities were found up to 500 km into the ice edge in all concentrations of ice. Very low numbers of AMWs were found in the ice‐free waters of the West Antarctic Peninsula (WAP). A consistent relationship between AMW distribution and sea ice concentration weakens the support for the hypothesis that varying numbers of AMWs in ice‐covered waters were responsible for observed changes in estimated abundance. The potential decline in AMW abundance stresses the need for conservation measures and further studies into the AMW population status. Very low numbers of AMWs recorded in the ice‐free waters along the WAP support the hypothesis that this species is strongly dependent on sea ice and that forecasted sea ice changes have the potential of heavily impacting AMWs.

## 1 INTRODUCTION

**Figure 1:**
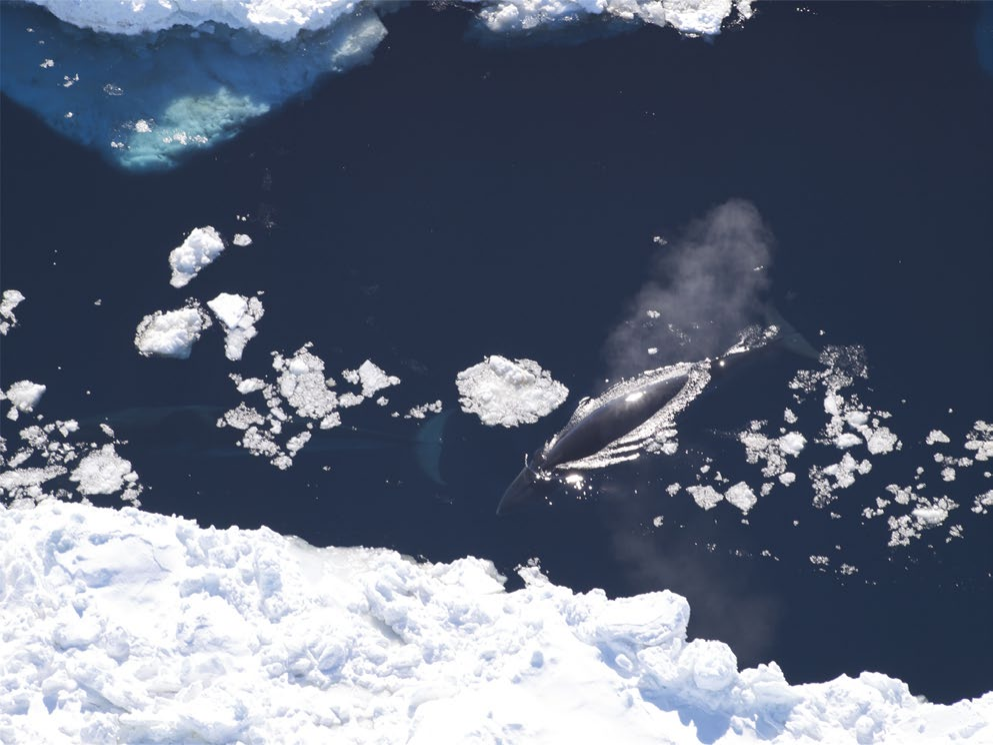
Two Antarctic minke whales in the sea ice‐covered waters of the Weddell Sea taking a breath in a narrow lead. Picture taken from the helicopter during a survey flight in February 2013. Photo: Helena Herr

Antarctic minke whales (*Balaenoptera bonaerensis*, AMW) are the only baleen whale species consistently inhabiting the ice‐covered parts of the Southern Ocean (Ainley, Dugger, Toniolo, & Gaffney, 2007). Highly adapted to sea‐ice habitats, they are frequently observed in a range of sea ice concentrations (Ainley et al., 2007, 2011, 2017; Scheidat et al., 2011; Thiele et al., 2004; Williams, Grand, et al., 2014). AMWs have been shown to feed on krill under the ice, with Friedlaender et al., (2014) suggesting that they exploit a unique niche among sympatric whale species feeding on krill in the Southern Ocean (Figure 1). Furthermore, predator avoidance has been suggested as another reason for AMWs to use sea‐ice habitats, inaccessible to Type A killer whales (Pitman & Ensor, 2003).

Despite sea ice having been recognized as an important habitat characteristic for a long time (Ainley et al., 2007, 2017; Ribic, Ainley, & Fraser, 1991), little is known about the variations in densities of AMWs in sea ice‐covered areas. Many Antarctic research voyages collecting information on cetacean distribution and abundance do not venture much inside the ice edge as either the research program is dedicated to mainly ice‐free waters (Santora, Schroeder, & Loeb, 2014), or ships are not ice‐strengthened and have to restrict their dedicated survey efforts to operationally safe, ice‐free waters (Murase et al., 2003). Even from icebreakers, conventional line‐transect surveys for density estimation are impossible on ships navigating or breaking through ice, constantly changing speed and direction (Ainley et al., 2011; Buckland et al., 2001). Moreover, biases in shipboard cetacean surveys in ice‐covered waters may arise from the influence of the ship's presence (e.g., its noise and the noise caused by ice‐breaking activities) on the detection rate of cetaceans due to behavioral reactions (Erbe & Farmer, 2000). Consequently, reliable information on densities of AMWs in sea ice is lacking.

This information, however, is crucial for assessing the species' vulnerability with regard to predicted, climate‐related changes in sea ice conditions (Silber et al., 2017). High‐latitude marine systems are among the regions responding most rapidly to changing climatic conditions, with ice‐obligate species facing some of the largest changes in their habitat (Nicol, Worby, & Leaper, 2008; Silber et al., 2017). Furthermore, knowledge about the order of magnitude of AMW numbers in sea ice‐covered areas could help gauge whether the proportion of the population inside the ice edge has the potential to account for at least some of the estimated decline in circumpolar AMW abundance between the late 1980s and the late 1990s (IWC, 2013). AMW abundance was last estimated at 515,000 (95% CI: 361,000–733,000) individuals based on data from the third and most recent circumpolar shipboard sighting survey (also known as CPIII; conducted under the auspices of the International Whaling Commission (IWC) during the austral summers of 1992/93 to 2003/04; IWC, 2013). Comparison with an abundance estimate of 720,000 (95% CI: 512,000–1,012,000) obtained from the second circumpolar survey (CPII; 1985/86‐1990/91) suggests a decline of approximately 30% between the late 1980s and the late 1990s (IWC, 2013). However, there is considerable uncertainty regarding the extent to which this reflects true population decline, distributional shifts or changing survey methods (IWC, 2013). Both abundance estimates are subject to some degree of negative bias since the full range of the species' summer distribution was not covered by the surveys (Branch & Butterworth, 2001). It is unclear, whether a shift between the two surveys in the proportion of the population that was north or south of the survey boundary, and therefore unavailable during the surveys, could be responsible for the observed change in estimated abundance. To the north, the circumpolar abundance estimates from CPII and CPIII surveys were restricted to the area south of 60°S, a latitude where density of AMWs is assumed to be low and the range begins to overlap with the dwarf minke whale (*B. acutorostrata*; Best, 1985). The southern boundary of both surveys was determined by the edge of the Marginal Ice Zone (MIZ), hereafter referred to as the ice edge. The proportion of the population of AMWs sampled may thus have been affected by intra‐ or inter‐seasonal changes in the position of the ice edge changing the area sampled and, ultimately, the numbers of animals encountered. Furthermore, any overall changes in the relative proportion of AMWs within ice‐covered areas throughout the years of the IWC's sighting surveys may have affected the number of whales available within the area up to the ice edge. These and other survey‐related hypotheses to explain the decline in estimated abundances are summarized in Murase and Bravington (2012). Ultimately, it remains unknown whether the number of AMWs in ice‐covered areas (i.e., unavailable to IWC shipboard surveys) had a large enough effect on the abundance estimates from open‐water to affect the conclusion that the population declined. If the decline is real, the causes are currently unknown, and the decline may be continuing. This raises concerns for the long‐term conservation of the species in the face of climate change, but also complicates the politically charged debate over whale management in the Southern Ocean, particularly Japanese special permit whaling (Brierley et al., 2016; Gales, Kasuya, Clapham, & Brownell, 2005).

Analyzing densities of AMWs in sea ice‐covered areas and variation therein is one way to test some of the hypotheses trying to explain the observed decrease in abundance. While it remains impossible to produce retrospective estimates of absolute abundance of AMWs in sea ice‐covered areas for the years that the IWC surveys were running, regional relative density estimates from ice‐covered parts of the habitat can provide an idea of likely magnitudes of abundances of minke whales inside sea ice regions (i.e., as compared to those found in adjacent open‐water areas) and potential variations therein. This may allow consideration whether the “moved‐into‐sea ice” hypothesis is at least tenable. Moreover, in the likely absence of absolute circumpolar abundance estimates in the near future, identifying dependencies and vulnerabilities of the species in their habitat seems to be a reasonable step toward helping to ensure protection, recognize threats, and mitigate impacts.

Aerial surveys have been proven as a means to survey ice‐covered areas at constant survey speeds, with assumed minimal disturbance effects on animal distribution and effectively collecting cetacean sighting data in 0%–100% ice concentrations (Herr et al., 2016; Scheidat et al., 2011; Vacquié‐Garcia et al., 2017; Williams, Kelly, et al., 2014). Williams, Kelly, et al. (2014) provided first information on relative AMW abundance on both sides of the ice edge, suggesting up to 20% of AMWs of the Weddell Sea were within ice‐covered waters.

In this study, we investigate AMW densities along gradients of sea ice concentration, based on five icebreaker‐supported helicopter surveys, including the two surveys evaluated by Williams, Kelly, et al. (2014). The much broader temporal and spatial extent of this data set allows us to not only quantify the numbers of AMWs within the ice‐covered areas but also to assess the spatial and temporal variability therein.

Here, we present the first distribution models for the density of AMWs based on the largest existing aerial survey data set from the Southern Ocean.

## 2 METHODS

### 2.1 Aerial surveys

We conducted aerial surveys during five expeditions of the German research icebreaker *Polarstern* (Alfred Wegener Institute, Helmholtz Centre for Polar and Marine Research, 2017) between 2006 and 2013 (ANT23‐8: Gutt, 2008; ANT25‐2: Boebel, 2009; ANT27‐2: Fahrbach, 2011; ANT28‐2: Kattner, 2012; ANT29‐3: Gutt, 2013; Table 1) using the two onboard helicopters of the type BO 105. For all five expeditions, the ship's track was designed for purposes of other research projects onboard and the dedicated helicopter surveys used the ship as a platform of opportunity along the given expedition route.

**Table 1:** Summary of effort and minke whale sightings for all five RV *Polarstern* expeditions

Expedition	Dates	Effort (km)	No. of sightings	No. of individuals	No. of sightings eligible for det. function modeling	No. of individuals in groups eligible for det. function modeling	Reference & data accessibility
ANT23−8	23 Nov 2006 –30 Jan 2007	12,903	71	158	42	96	Scheidat and Herr (2018); https://doi.pangaea.de/10.1594/PANGAEA.894937
ANT25−2	5 Dec 2008 –5 Jan 2009	6,182	22	26	11	13	Scheidat, Herr, and Siebert (2018); https://doi.pangaea.de/10.1594/PANGAEA.894936
ANT27−2	28 Nov 2010 –5 Feb 2011	13,206	44	69	27	41	Herr, Lehnert, and Siebert (2018a); https://doi.pangaea.de/10.1594/PANGAEA.894934
ANT28−2	3 Dec 2011 –5 Jan 2012	772	0	0	0	0	Herr, Lehnert, and Siebert (2018b); https://doi.pangaea.de/10.1594/PANGAEA.894924
ANT29−3	22 Jan 2013 –18 Mar 2013	7,632	18	33	15	29	Herr and Siebert (2018); https://doi.pangaea.de/10.1594/PANGAEA.894914
Sum		40,695	155	286	95	199	

Note

Total numbers of sightings are given along with numbers of sightings that were used in the detection function modeling process. Excluded from detection function modeling were sightings that a) were made by the right‐hand side observer and b) occurred under poor sighting conditions.

**Figure 2:**
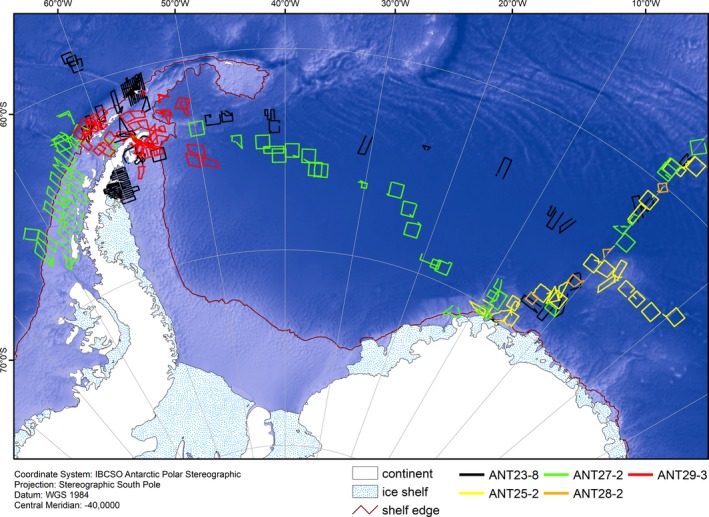
Tracks of all helicopter survey flights conducted during five expeditions of RV *Polarstern* between 2006 and 2013 (see Table 1 for expedition dates). The red line delineates the Antarctic shelf break as detected in this study. Depicted depth is based on the ETOPO grid (Amante & Eakins, 2009)

Given logistical constraints and daily changes in sea ice conditions, it was impossible to  create a systematic survey design in advance of the survey. This meant that the study was designed at the outset in anticipation of the use of model‐based density/abundance estimators over conventional/design‐based analyses (Buckland et al., 2007; Hedley & Buckland, 2004; Miller, Burt, Rexstad, & Thomas, 2013). We planned all flights in an *ad‐hoc* manner when weather conditions and ship's logistics permitted. Track lines were designed around the current position of RV *Polarstern*, maintaining basic principles of survey design following Buckland et al. (2001), such as random choice of starting points of transects and corresponding random placement of transects. Covered track lines are shown in Figure 2. All survey flights were conducted following line‐transect distance sampling methodology (Buckland et al., 2001) at a constant altitude of 600 feet (183 m) and a speed of 80–90 knots (145–165 km/h). Two observers seated in the back of the helicopter observed the area to the right and to the left side of the helicopter, respectively. As the helicopters were not equipped with bubble windows, the observers in the back were not able to observe the area directly under the helicopter, blocking from view approximately 80 m to each side of the transect line. A third observer was seated in the left front seat of the helicopter, which allowed a direct view onto the transect line through the front bottom window of the helicopter. Together, the left and front observers were able to provide full coverage of the left side of the transect line. Only these data, from the completely surveyed left side of the helicopter, were later used in detection function modeling.

All data were entered directly into a computer continuously storing GPS data obtained by a handheld GPS device in intervals of four seconds. Environmental and sighting conditions (sea state, cloud cover, glare, ice coverage, subjective sighting condition [a compound variable which describes the overall ease of observing minke whales, dependent on weather and ambient light conditions, taking three levels: “good”, “moderate” and “poor”]) were entered as assessed by the observers. Sightings were recorded noting species, distance to transect line (calculated postsurvey via declination and horizontal angle), and group size. Declination angles were measured using inclinometers. If a sighting occurred and the species could not be identified or group size could not be determined immediately, the survey was halted in order to approach the sighting for closer inspection, a procedure known as “closing mode” (Calambokidis & Barlow, 2004; Strindberg & Buckland, 2004). After identification, the helicopter returned to the transect line at the point of departure and the survey was resumed.

### 2.2 Data preparation

All survey data were associated with the following parameters: day‐specific ice cover derived from satellite data and distance to the ice edge (i.e., edge of MIZ), distance to shelf break, water depth, and bathymetric slope. Therefore, required data were obtained as described in the following.

#### 2.2.1 Sea‐ice cover and distance to ice edge

The daily 6.25 km resolution images of ice concentration using the ASI algorithm on data from the Advanced Microwave Scanning Radiometer for EOS (AMSR‐E) and AMSR2 satellite sensors from the Institute of Environmental Physics, University of Bremen, https://seaice.uni-bremen.de/data/amsre/ (Spreen, Kaleschke, & Heygster, 2008), were used to extract ice concentration values for each survey point. The same data source was used to estimate the position of the outer boundary of the MIZ for each survey day and subsequently calculate the distance to the ice edge for every data point. The ice edge was defined as the 15% contour of ice concentration (a typical threshold used to define the ice edge within satellite sea‐ice data; Worby & Comiso, 2004), that is a smoothed line bounding the 15% ice concentration margin. We used built‐in “Spatial Analyst” functions in ArcMap (Environmental Systems Research Institute, Inc., Redlands, California, 2009) to select the largest polygon of contiguous raster pixels featuring at least 15% ice concentration, defining the outermost edge, and then smoothing it using the “Boundary Clean” function. Finally, we calculated the Euclidean distance (in km, projection: Stereographic South Pole) to the closest ice edge position of the respective day for each recorded effort position (recorded every 4 s), cetacean sightings and all midpoints of the prediction grids, differentiating between distances inside and outside of the ice edge.

#### 2.2.2 Depth and slope

Depth and bathymetric slope (rate of change in depth over distance) values were extracted from the GEBCO 2014 grid available from the British Oceanographic Data Centre (BODC). We calculated slope values using the ArcMap “Slope” tool (Burrough & McDonell, 1998). Depth and slope values were extracted for each survey point as well as the prediction grid.

#### 2.2.3 Distance to shelf break

The shelf break is defined as the line between the shelf and the upper continental slope. It is generally represented by a relatively abrupt increase in seabed inclination from the flat shelf (<1°) to the steeper upper continental slope (>1°). Globally, the shelf break generally is between 100 and 150 m water depth (Weatherall et al., 2015). Around Antarctica, the ice load and the resulting isostatic equilibrium (Ivins & James, 2005; Whitehouse, Bentley, Milne, King, & Thomas, 2012) and erosion (Livingstone et al., 2016) result in a deep shelf. There, the shelf break is mostly located between 400 and 600 m water depth (Arndt et al., 2013).

**Figure 3:**
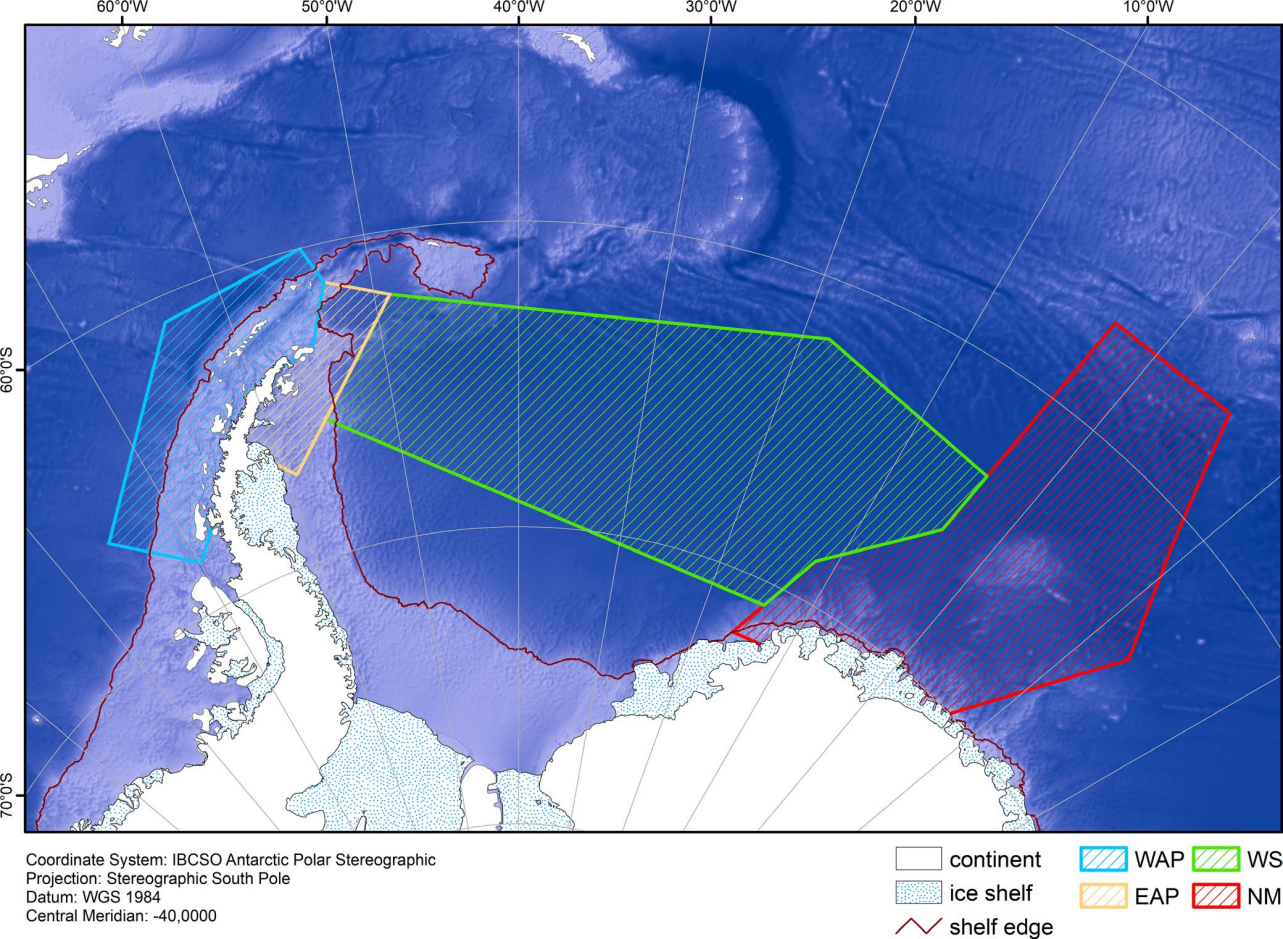
Strata designed postsurvey for density prediction and comparison; WAP = West Antarctic Peninsula, EAP = East Antarctic Peninsula, WS = Weddell Sea, and NM = Neumayer. The red line delineates the Antarctic shelf break as detected in this study. Depicted depth is based on the ETOPO grid (Amante & Eakins, 2009)

For this study, the shelf break (Figure 3) was detected on the base of the International Bathymetric Chart of the Southern Ocean (IBCSO) (Arndt et al., 2013). From the IBCSO raster data set, we calculated a slope angle raster data set following the approach of Burrough and McDonell (1998). The 1° slope angle line in most places clearly indicates the shelf break. In areas where the slope angle was less distinct, also the 500 m water depth contour and the hillshade relief raster data set (an illuminated terrain model generating a pseudo 3‐D visualization) were used to define the shelf break. North of the Antarctic Peninsula, the shelf break was extended from the Antarctic Peninsula to the South Orkney Plateau although the seabed dropped to around 1,000 m water depth in places. We considered the Bransfield Strait a tectonic inner‐shelf depression and defined the shelf break west of the South Shetland Island. In the southern Weddell Sea, sounding data were sparse, and IBCSO was often based on satellite altimetry data (Arndt et al., 2013). The lower resolution of these data sometimes reduced the accuracy of the detection of the shelf break.

Distance to the shelf break was then calculated (in Stereographic South Polar projection) for each data point, discriminating between positions on versus off the shelf.

### 2.3 Data analyses

#### 2.3.1 Detection function

We used distance sampling methods to estimate the probability of detection as a function of distance from the track line (Buckland et al., 2001). As mentioned above, we only used data from the left side (collected by the left and front observers) for modeling a detection function. In order to ensure adequate sample size, we pooled the sightings, and all their associated covariates, from all survey years, to estimate a single detection function. All minke whale sightings were used, including *B. bonaerensis*, *B. acuturostrata,* and sightings that could not be clearly identified as either of them or that were defined as “minke‐like”. As the front observer had an unobstructed view of the track line, no left truncation was applied to the perpendicular distances going into the fit of the detection functions.

Half‐normal and hazard‐rate detection function models were tested (Buckland et al., 2001) using the software package “mrds” (Laake, Borchers, Thomas, Miller, & Bishop, 2013) in R Version 3.0.1 (R Core Team, 2013). A multiple covariate distance sampling (MCDS) model framework was used to estimate the detection function (Marques & Buckland, 2004), with the effect of covariates entering via the scale parameter of the function, with the assumption that detection at the track line is certain (i.e., *g*(0) = 1). Covariates tested in the MCDS component, in addition to perpendicular distance, included sighting condition (“good” or “moderate”; any sightings recorded in “poor” sighting conditions were removed from analysis), Beaufort sea state, group size and a measure of local sea ice concentration (ice concentration as judged by the observers), which was classified as 0%–9% ice coverage = “no ice” and 10%–100% = “ice”. Ice concentration was included as a factor to test for evidence that increasing complexity in the visual field may decrease the probability of detection. Perpendicular distances were truncated to exclude the furthest ~10% of detections, which is recommended to avoid overfitting the tail of the distribution (Buckland et al., 2001). The best detection function model was selected based on Akaike's information criterion (AIC; Akaike, 1974).

#### 2.3.2 Density surface model

A density surface modeling approach was used to produce predictions on how the density of AMWs varied over space and time, and with environmental covariates. Note that these density estimates could not be corrected for availability bias (sensu Marsh & Sinclair, 1989) and it was thus assumed that *g*(0) = 1 (Buckland et al., 2001). This means, that estimated densities are minimum densities and most likely an underestimate. We used the count method, as described by Hedley and Buckland (2004) and Miller et al. (2013), in combination with generalized additive models (GAMs; Wood, 2006), to predict densities of AMWs across the study area. Restricted maximum likelihood was used as the criterion for estimating smoothing parameters (Wood, 2011). The response variable was the number of individuals per “segment” of transect, where the segment length was selected to ensure relative homogeneity in sighting conditions within a single segment. Based on analyses of varying segment lengths by exploration of the dispersion patterns of whale detections and of the spatial autocorrelation of environmental covariates across the segments, a segment length of 30 km was selected in order to balance between over‐dispersion in the number of animals sighted and having too much heterogeneity in sighting conditions within a single segment. Effort data with sighting conditions less than “moderate” (i.e., poor) in quality were removed from the analysis. An offset variable was incorporated in the model to account for changes in estimated probabilities of detection within each segment, which ultimately manifest in differences of areas effectively searched within the segments (Miller et al., 2013). The log‐transformed effective search area, estimated using the MCDS model described above, was used as the offset in the GAM‐based density surface model.

The modeling for the density surfaces was undertaken in R (R Development Core Team, 2014) using the mgcv package (mgcv v1.7–28; Wood, 2006, 2011). A Tweedie distribution (Jørgensen, 1987) was used to model the count data (where the Tweedie parameter was estimated during the GAM fit). Variance from the detection function fitting was propagated through the GAMs at the segment level, to the final abundance estimates via a random effects method described in Williams et al. (2011) and Miller et al. (2013). Variables tested for inclusion in the spatio‐environmental models included smooths of *x* and *y* (projected longitude and latitude values; Lambert azimuthal equal area projection), water depth, bathymetric slope, distance to the shelf break, ASI sea ice concentration (corresponding to dates of particular survey effort), and distance to the ice edge (derived from the satellite data for each survey day as described above). Survey season was included in the density surface models as a factor term to account for any inter‐seasonal variations that may occur in AMW densities across the survey areas.

#### 2.3.3 Prediction

We predicted and compared AMW densities between four strata: Neumayer (NM), Weddell Sea (WS), East Antarctic Peninsula (EAP), and West Antarctic Peninsula (WAP) (Figure 3). The delineation of the strata was (a) based on spatial and temporal distribution of survey effort (i.e., with the aim to limit the amount of extrapolation, both in space and time) and (b) aimed at a representation of ecologically meaningful spatial units, especially with regard to average ice concentrations: In particular, WAP comprises the survey area covered west of the Antarctic Peninsula, representing habitat with less ice than the remainder of the survey area; EAP represents a coastal area on the shelf with very variable ice conditions; WS comprises the waters of the Weddell Sea with usually high ice coverage; and NM represents the waters east of the Weddell Sea, with very variable northerly extension of the MIZ.

**Figure 4:**
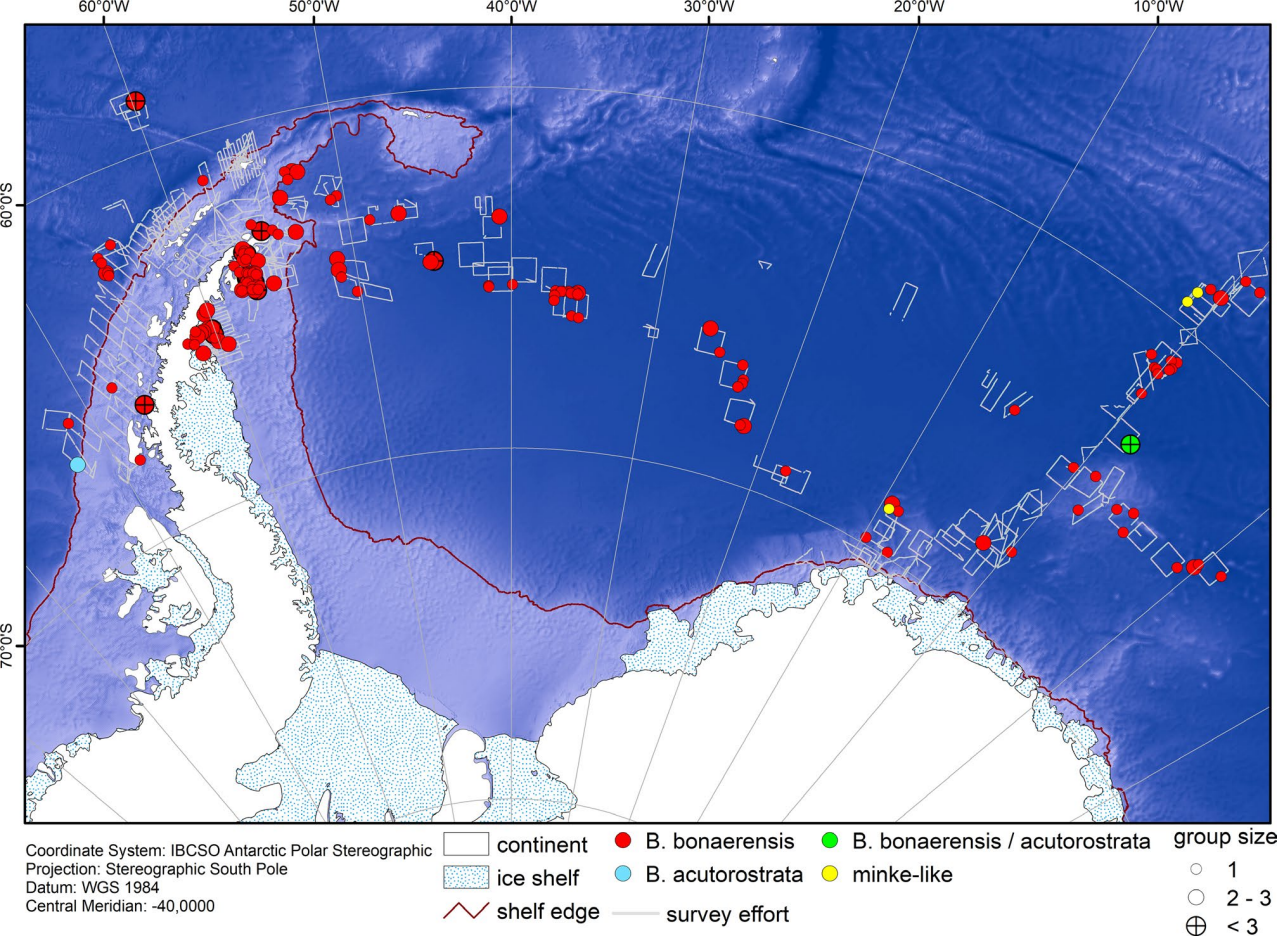
Positions of all minke whale sightings from aerial surveys of five RV *Polarstern* expeditions (see Figure 2). Sightings include Antarctic minke whale *Balaenoptera bonaerensis*, dwarf minke whale *B. acutorostrata*, uncertain identification of either *B. acutorostrata* or *B. bonaerensis* and “minke‐like” sightings. Gray lines indicate position of survey effort; red line delineates shelf break as detected in this study. Depth based on the ETOPO grid (Amante & Eakins, 2009)

All areas were visited by at least two expeditions thus providing some temporal coverage within each stratum. For each stratum, we produced a prediction grid. The spacing of the prediction grid was 6.25 km, representing the resolution of the coarsest environmental variable, i.e., the satellite‐based ASI ice concentration data). The prediction grids contained information on water depth, bathymetric slope, daily sea ice concentration, distance to ice edge, and distance to shelf break for each grid point, in addition to projected coordinates. We predicted AMW densities for each stratum for selected dates of the survey periods, meeting approximately the middle of the corresponding effort periods of the respective surveys that visited the stratum, to account for extant sea ice conditions.

## 3 RESULTS

In total, 40,695 km of track lines were covered by helicopter surveys (Figure 2, Table 2). Flights were conducted on 106 survey days, with concentration of effort in December and January, across various austral summer seasons (Appendix Table A1) and varying sighting conditions (Appendix Tables A2, A3 & A4). 25,987 km of survey effort were allocated to EAP, WS and NM, covering areas of 0%–100% of ice concentration in good and moderate conditions. Almost half of the survey effort (18,077 km) in EAP, WS and NM was allocated to areas inside the ice edge. In WAP, 13,631 km of survey effort was completed in mainly ice‐free waters (Appendix Table A5).

**Table 2:** Summary of total effort and minke whale sightings per stratum plus encounter rates

Stratum	Area (km^2^)	Survey effort (km)	No.of sightings	No.of individuals	Encounter rate groups/km
NM	1,150,664	11,607	31	42	0.003
WS	1,579,102	7,213	34	48	0.005
EAP	145,820	7,619	74	161	0.010
WAP	425,938	13,631	15	30	0.001
n.a.	n.a.	625	1	5	0.002
Total	3,273,907	40,695	155	286	0.003

**Table 3:** Estimates of densities (individuals/km^2^) of Antarctic minke whales for separate strata and for dates representing approximately the midpoint of the associated survey effort (uncorrected for *g*(0) and availability bias)

Stratum	Date	Stratum area (km^2^)	Mean density (ind./km^2^)	CV
NM	05. Dec 2006	1,150,664	0.0027	0.31
NM	19. Dec 2008		0.0024	0.33
NM	18. Dec 2010		0.0040	0.24
WS	15. Dec 2006	1,579,102	0.0045	0.21
WS	30. Dec 2010		0.0043	0.25
EAP	14. Jan 2007	145,820	0.0131	0.22
EAP	11. Feb 2013		0.0082	0.25
WAP	n.a.	425,938	0.0006	0.39

Note

Strata: NM = Neumayer, WS = Weddell Sea, EAP = East Antarctic Peninsula, WAP = West Antarctic Peninsula. For WAP no ice edge could be detected, thus, no model predictions were possible. Density given for WAP is based on conventional distance sampling analysis.

A total of 155 minke whale sightings, comprising 286 individuals including one calf, were recorded (Tables 1 and 2, Figure 4). AMWs (*B. bonaerensis*) represented the majority, with 150 sightings of 275 individuals. One sighting of two individuals was identified as *B. acuturostrata*, one sighting of six individuals as either *B. bonaerensis* or *B. acuturostrata,* and three sightings of single animals were recorded as “minke‐like” (Figure 4). We encountered AMWs all over the study area. However, encounter rates differed greatly between strata. The lowest encounter rate (0.001 groups/km) was found in WAP (west coast of the Antarctic Peninsula), the highest encounter rate (0.010 groups/km) was found in EAP (east coast of the Antarctic Peninsula). East of EAP, a longitudinal gradient was observed with encounter rates generally decreasing from west to east (Table 2).

Antarctic minke whales were encountered in sea‐ice covers of up to 99% of the visual field of the observers / 100% ice concentration as indicated by the ASI sea‐ice data and up to 981 km inside the ice edge. The maximum distance inside the ice edge covered by aerial surveys was 1,192 km.

### 3.1 Detection function

**Figure 5:**
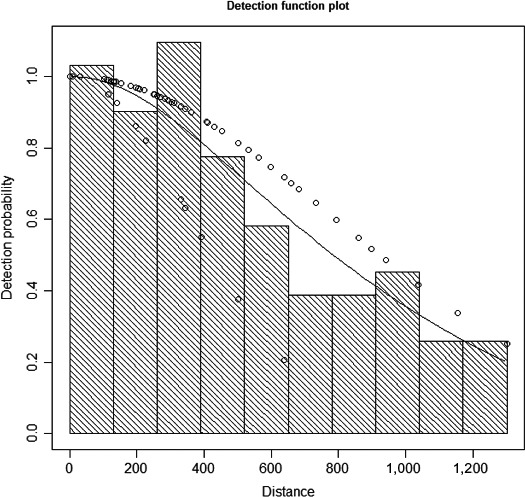
Distribution of minke whale sightings at various distances (*x*‐axis, [m]) from the track line, pooled across all surveys. The selected detection function (solid line) was a half‐normal function scaled by a sighting condition score (moderate and good). Circles are the probability of detection for each sighting given its perpendicular distance and other covariate values. The series of open circles above the line shows the detection function under *good* sighting conditions (effective strip half‐width, ESW = 887 m), whereas the circles below the line show the detection function under *moderate* conditions (ESW = 450 m)

Of all recorded sightings, 105 sightings of 199 individuals (104 sightings of *B. bonaerensis*, 1 sighting of *B. acuturostrata*) were eligible for detection function modeling (Table 1). Detection distances were right‐truncated at 1,300 m for fitting the detection function. After comparing the AIC from all permutations of the detection functions and potential scaling variables, the model with lowest AIC was selected, which was a half‐normal detection function with a distance sampling model scaled by sighting condition score (1 = moderate, 2 = good) as the most parsimonious model (Figure 5). The estimated mean detection probability between track line and the truncation distance was 0.61 (CV = 0.10). Moving between a sighting condition score of moderate to good nearly doubled the effective strip half‐width (i.e., from 450 to 887 m). Details on the distribution of effort over sighting conditions is provided in the Appendix (Tables A2, A3, A4). Of the total effort, 39,526 km (97%) were surveyed under “good” or “moderate” sighting conditions.

### 3.2 Density surface model

**Figure 6:**
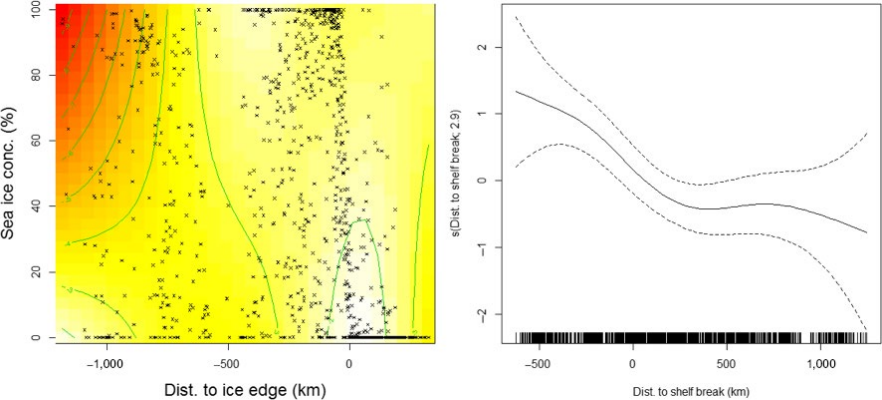
Plots representing smooths from the final density surface model describing densities of Antarctic minke whales. Left: plot of the tensor product smooth of distance to ice edge and sea ice concentration. Negative values for distance to ice edge indicate regions inside the ice edge; positive values are outside. Black crosses indicate sampling positions; red indicates lower whale densities, white the highest predicted densities (predictions on a log scale). Right: plot showing the smooth function of distance to shelf break. Dotted lines show the ~95% confidence interval for the smooth term. *Y*‐axis is on a log scale. Number in bracket represents the effective degrees of freedom of the smooth term. Marks and ticks at *x*‐axes represent the sampling locations

A density surface model composed of a combination of a tensor product of ASI sea ice concentration and distance to ice edge and a smoother of distance to shelf break was selected as the best GAM to describe the distribution of densities of AMWs (deviance explained of 20.9%; Figure 6). The GAM results show a combined influence of ice concentration and distance to ice edge on the densities of AMWs. Highest minke whale densities were predicted around the ice edge at low to medium ASI sea ice concentration, with a peak in AMW densities at the ice edge. Moving into the ice, these densities remained fairly constant at a medium range up to 500 km beyond the ice edge, in all concentrations of ice. Lowest densities were found far away from the ice edge in areas of high ice concentration. At the same time, areas far away from the ice edge with lower ice concentration show medium densities of AMWs. A gradient of minke whale densities shows highest densities on the shelf and decreasing densities with distance from the shelf. Distribution of ice concentration in relation to distance from the ice edge is given in the Appendix (Figure A1).

Based on the best model, we predicted AMW densities (uncorrected for availability) for separate strata and specific dates (to reflect respective ice conditions). No predictions using this model were possible for WAP, as no ice edge could be identified in this stratum for any year. Low ice concentrations in this area during all five survey years resulted in no MIZ; geographically, the closest ice edge was located east of and, thereby, “behind” the Antarctic Peninsula. We therefore considered distances to this ice edge as not ecologically meaningful for use in the model. The sample size (i.e., the number of sightings) in WAP, however, was too small to create an independent GAM solely for WAP, based on the remaining variables. In order to obtain density predictions for this stratum in absence of a model‐based density estimate, we used conventional distance sampling (i.e., a simple detection function with no sighting covariates in the scale parameter, and a design‐based approach to estimating animal density), using the software package “Distance” (Miller, 2015) in R (R Core Team, 2015). WAP provided reasonably representative coverage by surveyed track lines for conventional distance sampling analyses (WAP stratum boundary is given in Figure 3 and realized effort is given in Figure 4), so that this approach seemed justifiable to obtain at least a rough density estimate for comparison with the other strata. The density estimated for WAP was 0.0006 ind./km^2^ (CV 0.39, 95% CI: 0.0003–0.0012).

**Figure 7:**
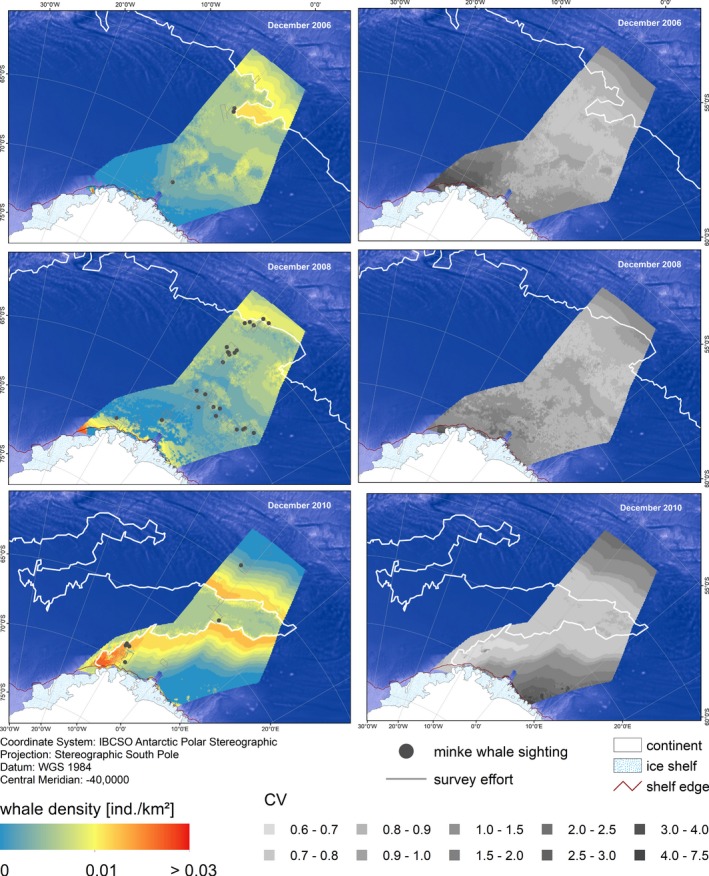
Predicted Antarctic minke whale densities and associated CVs in stratum NM (Neumayer) for specific dates based on the density surface model comprising a tensor product of ice concentration and distance to the ice edge and a smoother of distance to the shelf break. The white line indicates the position of the day‐specific ice edge: 05 December 2006, 19 December 2008, and 18 December 2010. Depicted depth is based on the ETOPO grid (Amante & Eakins, 2009)

**Figure 8:**
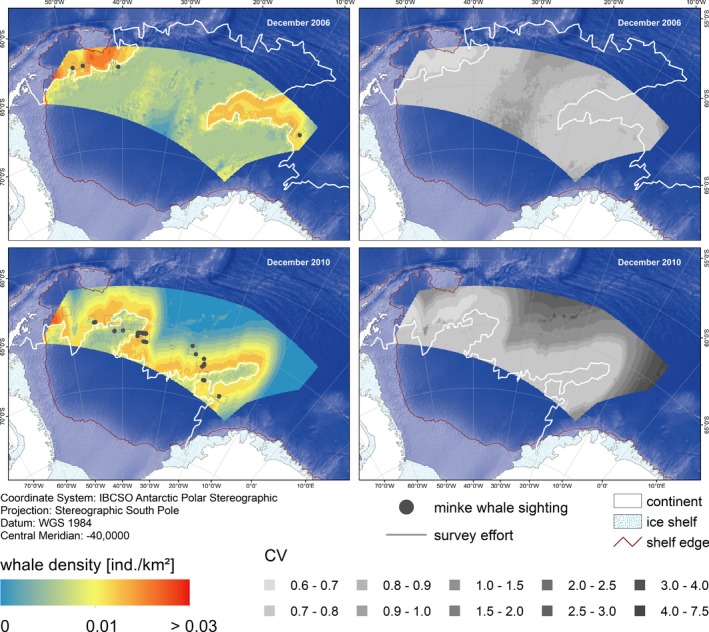
Predicted Antarctic minke whale densities and associated CVs in stratum WS (Weddell Sea) for specific dates based on the density surface model comprising a tensor product of ice concentration and distance to the ice edge and a smoother of distance to the shelf break. The white line indicates the position of the day‐specific ice edge: 15 December 2006 and 30 December 2010. Depicted depth is based on the ETOPO grid (Amante & Eakins, 2009)

**Figure 9:**
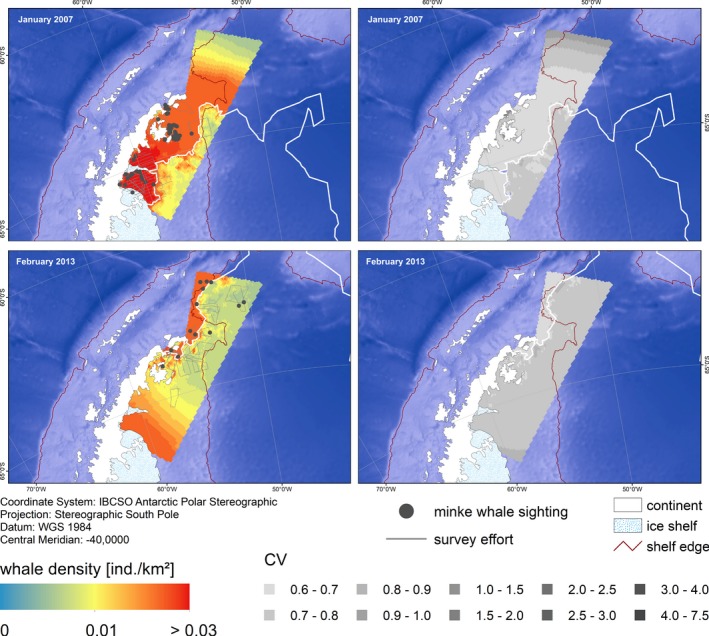
Predicted Antarctic minke whale densities and associated CVs in stratum EAP (East Antarctic Peninsula) for specific dates based on the density surface model comprising a tensor product of ice concentration and distance to the ice edge and a smoother of distance to the shelf break. The white line indicates the position of the day‐specific ice edge: 14 January 2007 and 11 February 2013. Depicted depth is based on the ETOPO grid (Amante & Eakins, 2009)

Predictions of AMW densities in EAP, WS, and NM showed a high variability over space and time (Figures 7, 8, 9). Predicted densities varied strongly between strata, with overall highest densities found in stratum EAP and overall lowest in NM. Within each stratum, predicted densities differed greatly between dates chosen for prediction, depending on the respective ice concentration therein. In EAP, densities ranged from 0.0082 (CV 0.25) to 0.0131 (CV 0.22) ind./km^2^, in NM from 0.0024 (CV 0.33) to 0.0040 (CV 0.24) ind./km^2^ (Table 3).

In Table 4, densities for ice‐covered and open‐water parts of respective strata are given. Mean densities in ice‐covered parts of the strata ranged between 0.0021 (CV 0.32) in NM and 0.0121 (CV 0.21) in EAP. Densities for open‐water areas were generally higher ranging from 0.0039 (CV 0.28) in NM to 0.0150 (CV 0.27) in EAP.

**Table 4:** Density of Antarctic Minke whales in open waters and inside the ice edge for separate strata and dates

Stratum	Date	Area (km^2^) open waters	Density (ind./km^2^) open waters	CV_open_	Area (km^2^) beyond ice edge	Density (ind./km^2^) beyond ice edge	CV_ice_
NM	05.12.2006	188,320	0.0057	0.56	962,344	0.0021	0.30
	19.12.2008	90,508	0.0055	0.71	1,060,156	0.0021	0.32
	18.12.2010	857,304	0.0039	0.28	293,359	0.0043	0.26
WS	15.12.2006	310,859	0.0088	0.23	1,268,242	0.0035	0.26
	30.12.2010	1,305,000	0.0042	0.28	274,102	0.0051	0.27
EAP	14.01.2007	104,922	0.0149	0.25	40,898	0.0086	0.26
	11.02.2013	14,531	0.0154	0.27	131,289	0.0074	0.28
WAP	n.a.						

## 4 DISCUSSION

Our results confirm that AMW distribution is strongly dependent on the position of the ice edge. This relationship was constant throughout the study period, with the highest densities of AMWs observed around the ice edge, decreasing gradually to both sides of the ice edge, with a steeper gradient in the ice compared to open water. Furthermore, we identified a relationship between densities of AMWs and satellite‐derived sea ice concentration, showing that, despite occurrence throughout the full range of ice concentrations, AMW densities are generally lower in high ice concentrations. Based on data obtained from aerial surveys covering the longitudinal extent between the 0° and the WAP, spanning a time series of 7 years and effort in 0%–100% of sea ice concentration, this result is the strongest signal from the currently most extensive data set on AMW density along gradients of sea ice concentration.

Apart from a strong association with the sea ice edge, we also found a relationship of AMW densities in relation to the distance to the shelf break, with higher densities on the shelf and decreasing densities with increasing distance from the shelf. This result is in line with previous studies who found distance to the Antarctic shelf break front (ASBF, Ainley et al., 2011); known also as the Antarctic slope front (Heywood et al., 2014)) or the position of the shelf break (Thiele et al., 2004) important factors describing AMW occurrence. A common observation is higher densities of Antarctic krill—a dominant prey item for AMWs (Friedlaender et al., 2006)—in the vicinity of the shelf break area in various locations throughout the Southern Ocean (Siegel & Watkins, 2016). These studies, however, had some difficulties separating the influence of the position of the shelf break from the ice edge, for example, in the Amundsen and Bellingshausen Seas, where these two factors mostly coincide during summer (Ainley et al., 2011). Our surveys, covering the Weddell Sea, where even in summer the sea‐ice extent largely exceeds the area of the shelf, suggest both parameters go some way to explain variation in AMW distribution. Highest densities of AMWs were predicted in areas where the ice edge occurred on or close to the shelf. However, in WAP, where no ice edge was present, AMW encounter rates on the shelf were low, relative to other areas surveyed in this study.

With regard to observed changes in AMW abundance between the IWC circumpolar abundance surveys CPII in the late 1980s and CPIII in the late 1990s, our results provide no evidence for highly variable AMW numbers residing in the ice‐covered waters. Based on our data, and with the year term dropping out of the model, the link between AMW densities and sea ice concentration appears to be constant throughout the time of this survey series, weakening evidence in support of the hypothesis that higher numbers of AMWs in the ice during the 1990s, as opposed to the 1980s, are responsible for the observed changes in population estimates. However, the relationship of AMWs and sea ice found in this study cannot exclude the possibility that long‐term changes in the behavior of AMWs took place before this series of aerial surveys and that AMWs used to inhabit the sea ice at lower densities prior to the time of CPIII.

Apart from the relationship of AMW densities and gradients in concentrations of sea ice and the consistency of this relationship over the years of the observation, our surveys provide further insights into potential sources of bias discussed in relation to the interpretation of the abundance comparisons between CPII and CPIII. Branch & Butterworth (2001) identified the fuzzy boundary between *B. acutorostrata* and *B. bonaerensis* around 60°S as one potential factor confounding trends. Helicopter surveys allow for a better discrimination between *B. bonaerensis* and *B. acutorostrata* than shipboard surveys due to the helicopter's ability to quickly close in on sightings for reliable species identification. Based on the ratios detected during our surveys (1 *B. acutorostrata* / 4 possible *B. acutorostrata* / 150 *B. bonaerensis* sightings), including latitudes up to 58°S, we feel comfortable downgrading that as a potential explanation of the change from CPII to CPIII, at least for the longitudinal range covered during our surveys.

Furthermore, inter‐(summer) seasonal longitudinal shifts have been discussed as potential for bias in the evaluation of abundances estimated for CPII and CPIII. Survey effort of IDCR/SOWER large‐scale surveys could only be achieved in relatively limited longitudinal extents each summer season, resulting in circumpolar assessments taking several years to complete. In our surveys, we detected a longitudinal gradient of AMW distribution, with no indication for large longitudinal shifts between survey years of our study (although the realized “design” of these aerial surveys is not necessarily optimal to test that hypothesis).

The results of our surveys highlight the importance of sea ice as a habitat feature for AMWs. Stratum WAP provided the lowest encounter rates of AMWs. Despite considerable survey effort (34% of the total effort across all survey seasons) realized in WAP, only 16 sightings of AMWs were recorded here, equaling <10% of the total number of sightings. Predictions by the selected density surface model were not possible for WAP, because the model included distance to ice edge as a covariate, which could not be determined in WAP. Here, no ice edge according to the definition of a line bounding the 15% ice concentration margin could be defined for any of the survey years in WAP. Density estimation by conventional distance sampling instead revealed 0.0006 ind./km^2^ (CV 0.39, 95% CI: 0.0003–0.0012). While this estimate is not directly comparable with the estimates for the other strata, it is valid to state that densities in WAP range in the order of a third of densities of all other strata (based on the assumption that availability bias for AMWs remains relatively constant throughout the strata). Our encounter rate (whale per surveyed km) is also substantially lower than those reported by Thiele et al. (2004) and Friedlaender et al. (2006). This may partly be attributed to lower availability during aerial surveys compared to shipboard surveys which observations by Thiele et al., (2004) and Friedlaender et al. (2006) were based on. Furthermore, minke whales are known to be encountered particularly in the embayments of the Antarctic Peninsula close to shore (Friedlaender et al., 2006; Williams, Hedley, & Hammond, 2006). These were covered by our surveys only to a limited extent, owing to an even survey coverage across the area. However, low encounter rates may also be related to the absence of ice in the area covered by our surveys, located further north around the tip of the Antarctic Peninsula compared to the other studies. In all our survey years, very low ice concentrations were encountered in stratum WAP. Observations from the Bellingshausen and Amundsen Sea have shown that more AMWs were observed in years with more ice compared to years with less ice along the Antarctic Peninsula (Kasamatsu, Ensor, Joyce, & Kimura, 2000; Thiele et al., 2004). Warmer sea‐surface temperatures, fewer cold water intrusions, and the smaller extents of sea ice have been suggested be related to the difference in distribution of minke whales along the Antarctic Peninsula (Kasamatsu et al., 2000). With sea ice providing habitat for the overwintering of phytoplankton and krill, seasons of high winter ice extension typically support greater primary production than seasons with less ice‐covered habitat in winter (Loeb et al., 1997; Meyer et al., 2017; Nicol et al., 2008). Overall winter sea‐ice extent has recently been suggested to directly affect the body condition of krill predators after the feeding seasons (Bengtson Nash et al., 2017; Braithwaite, Meeuwig, Letessier, Jenner, & Brierley, 2015; Seyboth et al., 2016). The Antarctic Peninsula region is undergoing rapid change, with increasing temperatures causing decreases in winter sea‐ice duration (Ducklow et al., 2007; Stammerjohn, Martinson, Smith, Yuan, & Rind, 2008; Turner, Maksym, Phillips, Marshall, & Meredith, 2013). The relatively low numbers of minke whales in this area may serve as a preview for potential climate change‐related effects on the species' distribution and abundance if the sea ice cover decreases around Antarctica. With the demonstrated importance of sea ice for AMWs, they are a primary candidate species for being dramatically affected by climate change‐related loss of sea ice.

The only confirmed sighting of *B. acutorostrata* occurred in WAP. High‐latitude records of *B. acutorostrata* are rare, with the species' center of distribution presumably located north of 60°S (Reilly et al., 2008). Multiple sightings of *B. acutorostrata* particularly at the Western Antarctic Peninsula (compiled in Acevedo et al., 2011) may serve as further indication of a warming WAP.

This study provides an important reminder that measuring densities is a powerful tool for inferring potential climate‐mediated shifts in distribution. Many climate change predictions rely on range maps to predict vulnerability (e.g., Lee, Maggini, Taylor, & Fuller, 2015; Ocampo‐Peñuela, Jenkins, Vijay, Li, & Pimm, 2016). But range boundaries can be fuzzy, fluid, or hard to survey, especially when depending on variable habitat qualifiers such as sea ice in this study. Our study could not have been accomplished with range maps alone, because range maps require a sometimes subjective decision to identify the point when extremely low density identifies the boundary between presence and absence. It can however be essential to determine even very low densities, local differences, and changes therein, when predicting responses of marine wildlife to climate change at the edge of their range (Williams, Grand, et al., 2014).

### 4.1 Error discussion

There are several sources of potential error associated with the results of this study. It has to be noted that encounter rates and densities in this study are minimum estimates, because no correction for availability or *g*(0) during the surveys could be undertaken. The relatively high survey speed during aerial surveys tends to increase availability bias, because animals have less time to appear in the field‐of‐view of observers, compared to shipboard surveys. Another concern, that ice cover could increase complexity in the visual field and thus influence detection probability of whales, was controlled by testing ice cover as a factor during detection function modeling. Ice cover dropped out as an explanatory variable, thus providing no evidence for ice cover impairing or enhancing the detectability of whales to the observers during these surveys. Unfortunately, there is no evidence to indicate how availability differs across the entire study region. Differences in availability between open water and ice‐covered areas may also apply, but again, there is no strong evidence to indicate the directions and magnitudes of such bias. We therefore decided not to apply a generic correction factor, but instead to use minimum density estimates as a baseline to reduce additional sources of error and assumption‐based conclusions. Clearly, it is important to begin estimating availability bias across a range of sea ice and open‐water habitats, to allow for corrected abundance estimates from aerial surveys. Current studies on AMW ecology by means of time‐depth recording tags (Friedlaender et al., 2014) provide insights into individual dive times, and the likely time animals spend across the gradients in sea ice concentrations and under the ice, preferred sea‐ice habitat and behavior in ice‐covered waters. In the long run, such data may help correcting for availability in a range of ice concentrations and open waters in order to produce estimates of absolute abundance from aerial surveys.

The position of the sea ice edge, as well as sea ice concentration, were derived from ASI satellite data. Herein lies another source of potential error in this study, due to the inaccuracy associated with these data sets (Zhao, Su, Stein, & Pang, 2015). For example, AMWs could not have been spotted in 100% of ice concentration (i.e., not visible to an observer). The ASI product does not discriminate between different thicknesses of ice. Very thin ice—physically not a hindrance for breathing for AMWs—could be identified as solid ice cover in a similar way as might a thick floe of ice. Moreover, the relatively coarse resolution of the used ASI product of 6.25 km may lead to generalizations. Also, the spatial and temporal variability of the ice concentration may introduce additional uncertainties. Ice drift can easily be in the order of a 6.25 km grid cell per day (Heil & Allison, 1999), while a daily product of satellite‐based sea ice concentration estimates can originate from overflights up to 24 hr apart. As no exact observation time is associated to each individual grid cell in the ASI sea ice concentration product, the surveys may originate from spatially and temporal shifted sea ice conditions compared to the ASI ice concentration product.

Finally, the results of this study apply to only one sector of the Southern Ocean. The observed longitudinal gradient across the Weddell Sea and the Antarctic Peninsula alone points to potential differences in AMW densities around Antarctica. In addition, sea ice conditions differ spatially as well, in addition to substantial variations throughout the summer season. While the WAP is seeing dramatic decreases in sea ice, other areas of the Antarctic are experiencing increases in winter sea‐ice cover (Parkinson & DiGirolamo, 2016). Therefore, information from other sectors of the Southern Ocean is necessary to complete the picture and to fully assess AMW relationships with sea ice.

## 5 CONCLUSION

This study increases and extends our knowledge about relative densities of AMWs in ice‐covered as well as open waters of the Southern Ocean. The multiyear data set provides indication for a consistent relationship of AMW distribution with the position of the sea ice edge and across a range of ice concentrations. Our results provide no indication for numbers of AMWs in ice‐covered waters highly variable in space and time. This weakens the support for the hypothesis that varying numbers of AMWs in ice‐covered waters were responsible for observed changes in AMW abundance between the two IWC circumpolar abundance surveys in the late 1980s and 1990s, respectively. The potential decline in AMW abundance stresses the need for conservation measures and further studies into the AMW population status.

Lowest densities of AMW observed in the areas with lowest ice concentration surveyed in this study (i.e., in WAP), are consistent with the hypothesis that this species is dependent on sea ice. The nature and magnitude of future responses of AMWs as a species are uncertain in novel environments as projected under climate change. An overall decrease of sea‐ice cover would narrow available habitat for AMWs. Our results suggest that the forecasted sea ice changes (Böning, Dispert, Visbeck, Rintoul, & Schwarzkopf, 2008; Rignot et al., 2008; Silber et al., 2017) have the potential of heavily impacting AMWs.

## CONFLICT OF INTEREST

None declared.

## AUTHORS CONTRIBUTION

MS, KHK, HH, NK, RW, and US conceived the idea for this study. MS, HH, LSL, and US designed the study. MS, LSL, KHK, RW, SV, and HH collected the cetacean survey data. HH and SV processed the cetacean survey data. BD processed and prepared the bathymetric data. MH processed and prepared the sea‐ice data. HH, NK, and SV analyzed and interpreted the combined data sets. HH conducted the GIS analyses, NK conducted the statistical analyses. HH drafted the manuscript. All authors critically revised the manuscript and gave their final approval for publication.

## Figures and Tables

**Figure 10:**
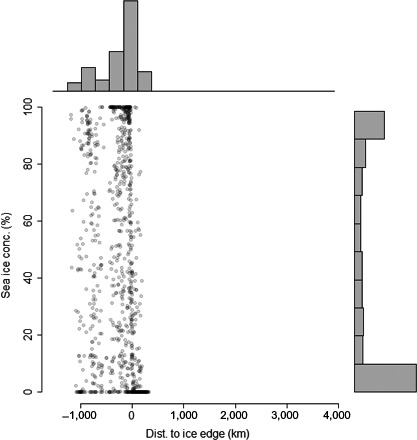
Distribution of distance to sea ice edge and ASI ice concentration across the EAP, WS, and NM. In WAP no ice edge could be detected

## Data Availability

All survey data used in this study are available from the Pangaea database: ANT23‐8: https://doi.pangaea.de/10.1594/PANGAEA.894937. ANT25‐2: https://doi.pangaea.de/10.1594/PANGAEA.894936. ANT27‐2: https://doi.pangaea.de/10.1594/PANGAEA.894934. ANT28‐2: https://doi.pangaea.de/10.1594/PANGAEA.894924. ANT29‐3: https://doi.pangaea.de/10.1594/PANGAEA.894914. Sea ice data are available from: https://seaice.uni-bremen.de/data/amsre/. Bathymetric data were derived from the International Bathymetric Chart of the Southern Ocean (IBCSO) available from the Pangea database: https://doi.org/10.1594/PANGAEA.805736

## APPENDIX 1

**Table A1:** Distribution of all effort over months

Month	Survey days	Effort (km)
December	47	18,065
January	40	15,601
February	13	4,763
March	6	2,266
Sum	106	40,695

**Table A2:** Distribution of effort over sighting conditions

Sighting condition	Effort (km)
good	31,629
moderate	7,897
poor	1,169
Total effort	40,695

**Table A3:** Distribution of effort over Beaufort sea states

Beaufort sea state	effort (km)
0	6,358.75
1	13,636.68
2	7,752.2
3	6,576.33
4	4,850.64
5	1,154.72
6	60.62
*n*.a.	305.14
Total effort	40,695

**Table A4:** Distribution of effort over ice conditions in good and moderate sighting conditions

Icecover in visual field	Effort (km) in good and moderate conditions
No ice (1%–9%)	20,868
Ice (10%–100%)	18,658
Total effort	39,526

**Table A5:** Distribution of effort between strata and allocation of effort with regard to ice edge in EAP, WS, and NM

Area	Effort (km)	Effort (km) under good and moderate conditions
NM, WS, EAP	26,608	25,987
Outside ice edge	8,257	7,910
Inside ice edge	18,351	18,077
WAP	14,087	13,539
Total effort	40,695	39,526
